# A Preliminary Validation of the Details of Function of Everyday Life Scale for the Detection of Early‐Stage Alzheimer’s Disease

**DOI:** 10.1002/alz.083768

**Published:** 2025-01-03

**Authors:** Clara Calia, Freddie O'Donald, Mario A Parra‐Rodriguez

**Affiliations:** ^1^ University of Edinburgh, Edinburgh United Kingdom; ^2^ NHS Tayside, Dundee United Kingdom; ^3^ Universidad Autónoma de Caribe, Barranquilla Colombia; ^4^ University of Strathclyde, Glasgow United Kingdom

## Abstract

**Background:**

Assessments for early‐stage Alzheimer’s disease (AD) aim to identify neuropsychological and functional impairments, which rarely correlate with early disease stages. We need to enhance our understanding of the cognitive aspects contributing to functional decline to improve sensitivity in functional assessment. The ability to bind information in memory declines in preclinical AD stages. However, it is unclear whether such cognitive deficits underlie functional impairment in prodromal AD stages. To explore this, the Details of Function of Everyday Life (DoFEL) has been developed, aiming to link the cognitive constructs of memory‐binding to specific impairments in activities of daily living.

**Method:**

The DoFEL underwent revision and latent structure exploration through Exploratory Factor Analysis in a non‐clinical sample (N = 559). This revised DoFEL was subjected to content validity review by eight dementia professionals, and factor structure was reassessed with Confirmatory Factor Analysis in a different sample (N = 135). Forty‐nine participants with MCI and thirty‐three healthy controls completed the DoFEL, Addenbrookes Cognitive Examination‐Revised (ACE‐R), and a Visual Short‐Term Memory Binding Task (VSTMBT). The correlation of revised DoFEL scores was examined in relation to ACE‐R and VSTMBT. Binomial regression was used to establish whether the revised DoFEL could differentiate healthy controls from MCI.

**Result:**

(1) Conjunctive and relational binding appeared to be represented as dimensions within DoFEL subscales. However, the factor structure was not replicated across all subscales, suggesting further scale revision would be of benefit. (2) Dementia professionals considered the revised DoFEL useful for detecting functional impairment in early‐stage AD. However, the relevance of some items to binding was questionable due to their broad nature. (3) Performance on the revised DoFEL (higher score indicating impairment) strongly correlated with performance on the ACE‐R, *r*(82) = ‐0.663, *p* < .001, and moderately correlated with performance on the VSTMBT, *r*(82) = ‐0.518, *p* = 0.003. (4) The revised DoFEL differentiated those on a healthy ageing trajectory from individuals diagnosed with MCI, demonstrating comparability with the ACE‐R.

**Conclusion:**

These findings offer initial evidence for the validity of the DoFEL. It indicates that the connection between binding and performance in functional activities could offer a valuable avenue for improving functional assessment during early‐stage AD.

